# Ischemic Strokes Due to Large-Vessel Occlusions Contribute Disproportionately to Stroke-Related Dependence and Death: A Review

**DOI:** 10.3389/fneur.2017.00651

**Published:** 2017-11-30

**Authors:** Konark Malhotra, Jeffrey Gornbein, Jeffrey L. Saver

**Affiliations:** ^1^Department of Neurology, West Virginia University, Charleston Division, Charleston, WV, United States; ^2^Department of Biomathematics, University of California Los Angeles Comprehensive Stroke Center, Los Angeles, CA, United States; ^3^Department of Neurology, University of California Los Angeles Comprehensive Stroke Center, Los Angeles, CA, United States

**Keywords:** ischemic, stroke, endovascular, vessel occlusion, morbidity

## Abstract

**Background:**

Since large-vessel occlusion (LVO)-related acute ischemic strokes (AIS) are associated with more severe deficits, we hypothesize that the endovascular thrombectomy (ET) may disproportionately benefit stroke-related dependence and death.

**Methods:**

To delineate LVO-AIS impact, systematic search identified studies measuring dependence or death [modified Rankin Scale (mRS) 3–6] or mortality following ischemic stroke among consecutive patients presenting with both LVO and non-LVO events within 24 h of symptom onset.

**Results:**

Among 197 articles reviewed, 2 met inclusion criteria, collectively enrolling 1,467 patients. Rates of dependence or death (mRS 3–6) within 3–6 months were higher after LVO than non-LVO ischemic stroke, 64 vs. 24%, odds ratio (OR) 4.46 (CI: 3.53–5.63, *p* < 0.0001). Mortality within 3–6 months was higher after LVO than non-LVO ischemic stroke, 26.2 vs. 1.3%, OR 4.09 (CI: 2.5–6.68), *p* < 0.0001. Consequently, while LVO ischemic events accounted for 38.7% (CI: 21.8–55.7%) of all acutely presenting ischemic strokes, they accounted for 61.6% (CI: 41.8–81.3%) of poststroke dependence or death and 95.6% (CI: 89.0–98.8%) of poststroke mortality. Using literature-based projections of LVO cerebral ischemia patients treatable within 8 h of onset, ET can be used in 21.4% of acutely presenting patients with ischemic stroke, and these events account for 34% of poststroke dependence and death and 52.8% of poststroke mortality.

**Conclusion:**

LVOs cause a little more than one-third of acutely presenting AIS, but are responsible for three-fifths of dependency and more than nine-tenths of mortality after AIS. At the population level, ET has a disproportionate benefit in reducing severe stroke outcomes.

Acute ischemic stroke (AIS) is a leading cause of morbidity and mortality worldwide (1). The advent of endovascular thrombectomy (ET) as a proven beneficial treatment for acute cerebral ischemia is a major advance in neurovascular therapeutics (2, 3). But concerns have been raised that treatment impact may be modest at the population level, as ET is appropriate only for the subset of patients with cerebral ischemia due to occlusions of large, proximal arteries that are accessible to thrombectomy devices.

Analyses of the potential general impact of ET have largely focused upon the proportion of patients treatable with the intervention and estimated at 4–18% of all ischemic stroke patients (4, 5). However, such analyses almost certainly underestimate the impact of thrombectomy treatment on stroke outcomes. Ischemic strokes due to large-vessel occlusions (LVOs), compared with non-LVO strokes, are larger in infarct size (6, 7), have more severe presenting deficits (8, 9), and have worse long-term outcomes (10, 11). LVO ischemic strokes, therefore, are likely to contribute disproportionately to population-level poststroke dependence and mortality, higher than their simple frequency of occurrence among acutely presenting patients. We, therefore, undertook a focused, meta-analytic review to delineate the frequency of LVO ischemic strokes and their relative contribution to stroke-related disability and death among all patients presenting with acute cerebral ischemia.

## Methods

To quantify the contribution of LVO ischemic strokes to poststroke severe functional outcomes, we systematically searched MEDLINE, PubMed, and Scopus databases to identify English language articles, published between January 1995 and September 2017, reporting, among consecutive acute cerebral ischemia patients, rates of: (1) LVO and non-LVO among all patients and (2) combined poststroke dependence and death [modified Rankin Scale (mRS) 3–6] separately among patients with and without LVO. The search strategy was reviewed by an expert medical librarian, and included the combination of MeSH terms and keywords: “stroke,” “cerebral ischemia,” “transient ischemic attack,” AND “vessel occlusion,” AND “modified Rankin scale,” “mRS,” or “Rankin scale.” We also performed a manual search of additional articles listed in the citations of retrieved and reviewed articles. Our study was exempt for approval from the Institutional Review Board of our Institution.

Retrieved articles were reviewed to determine if they met the following formal selection criteria: (1) adult patient population consisting of consecutive patients with AIS or acute cerebral ischemia (combined AIS and transient ischemic attack); (2) imaging of intracranial vessels performed early (within 24 h of onset) in all patients, using computed tomography angiography, magnetic resonance angiography, or catheter angiography; (3) rates of dependence or death 30–180 days after stroke onset reported separately among patients with and without LVO; (4) cohort collected predominantly prior to the advent of effective ET devices in 2012; and (5) study cohort size ≥50. Quality of fully eligible studies was assessed using the Newcastle–Ottawa Scale. When needed, we contacted authors of the original studies to clarify some aspects of previously reported data and to elicit any relevant unreported data.

To quantify the proportion of LVO-AIS patients treatable by ET within 8 h of onset (ETT8), we also performed systematic literature search to identify studies quantifying: (1) proportion of AIS with ED arrival within 6 h of onset (ED6) and (2) proportion of patients with stroke onset at a location within 60-min ambulance travel time (air or ground) to an endovascular center (AMB60) (permitting hospital arrival within 7 h after onset, allowing treatment within 8 h if door to puncture time is within 60 min); and (3) proportion of 7-h-arriving LVO patients who would not have any contraindications to ET, such as early hemorrhagic transformation, rapid progression to completed infarction, or poor vascular access (NoCONTR). Using these values, in addition to the pooled estimate of the fraction of all AIS due to LVO (FLVO), the fraction of all ischemic strokes that are treatable by ET within 8 h (ETT8) was then obtained by the formula: ETT8 = FLVO × ED6x AMB60 × NoCONTR.

Statistical analyses were carried out with STATA 14.2 (STATA Corp, College Station, TX, USA). As only two studies met selection criteria, heterogeneity was assessed by inspection rather than formal *I*^2^ statistic testing. In addition, assuming there would be at least some heterogeneity present, we computed all estimates and their 95% confidence bounds using a random-effects model (12).

## Results

Among 197 articles reviewed, 2 studies (11, 13) met selection criteria and collectively analyzed 1,467 patients (Table 1; Figure S1 in Supplementary Material). Both were of high quality, 9/9 on the Newcastle–Ottawa Scale (Table S1 in Supplementary Material) (14); both were multicenter studies from diverse regions, the US and Europe, and large, with 643 and 824 patients, respectively.

**Table 1:** Characteristics of included studies.

Study characteristics	Smith et al. (11)	van Seeters et al. (13)
Study enrollment	2006–2009	2009–2013
Region	United States, East and West Coast	Netherlands
Recruitment sites	2 University hospitals	6 Universities, 8 non-University hospitals
Study type	Prospective cohort	Prospective cohort
Cerebral ischemia type	AIS + TIA	AIS
Sample size	643/675^a^	824
Age, mean (SD)	68.6 ± 15.3	67.7 ± 14.1
Sex, female	48%	41%
NIHSS, mean (SD)	7.63 (±7.29)	7 (3–13)^b^
Vessel imaging modality	CTA	CTA
Study entry time window	<24 h of onset	<9 h of onset
Time from onset to vessel imaging	NR	118 min (median) (IQR 74–189)
Segments abstracted as large-vessel occlusions for this analysis	Supraclinoid ICA, M1, M2, BA, intracranial VA	Intracranial ICA, M1, P1, or BA
Timing of outcome modified Rankin scale assessment	180 days	90 days

*AIS, Acute ischemic stroke; CTA, Computed tomography angiography; NR, not reported; ICA, internal carotid artery; MCA: middle cerebral artery; PCA, posterior cerebral artery; M1, first MCA segment; M2, second MCA segment; VA, vertebral artery; BA; basilar artery; IQR; inter-quartile range*.

*^a^Study reported intracranial vessel imaging in 643/675 (95.2%) patients. Age, sex, and NIHSS data in this table are for the 675 patients; data in other tables are for the 643 with intracranial vessel imaging*.

*^b^Study reported NIHSS as median (IQR), while mean (SD) was not reported*.

In the model-based combined analysis of these two studies, among consecutive acute cerebral ischemia patients, LVO was present in 38.7% (CI 21.8–55.7%). Rates of dependence or death (mRS 3–6) within 3–6 months were higher after LVO than non-LVO ischemic stroke, 64% (CI: 60.0–68.0%) vs. 24% (CI: 21.3–26.8%), odds ratio (OR) 4.5 (CI 3.5–5.6), *p* < 0.0001. Consequently, while LVO ischemic events accounted for 38.7% of all acutely presenting ischemic strokes, they accounted for 61.6% (41.8–81.3%) of all poststroke combined dependence or death (Table 2).

**Table 2:** Rates and outcomes of large-vessel occlusion (LVO) and non-LVO acute cerebral ischemia.

	Smith et al. (11)	van Seeters et al. (13)	Pooled model-adjusted estimates
Study sample size	643	824	1,467
LVO, *n* (%)	328 (51.0%)	219 (26.5%)	38.7% (CI: 21.8–55.7%)

**Dependence and death (modified Rankin Scale 3–6)**			
Among all patients	280/643 (43.5%)	292/824 (35.4%)	39.4% (CI: 33.7–45.0%)
Among LVO	212/328 (64.6%)	138/219 (63.0%)	64.0% (CI: 60.0–68.0%)
Among non-LVO	68/315 (21.6%)	154/605 (25.5%)	24.0% (CI: 21.3–26.8%)
Odds ratio (OR), LVO vs. non-LVO	3.98 (2.86–5.54)	4.99 (3.59–6.94)	4.46 (CI: 3.53–5.63)
Fraction due to LVO	212/280 (75.7%)	138/292 (47.3%)	61.6% (CI: 41.8–81.3%)

**Mortality**			
Among all patients	90/643 (14.0%)	N/A	14.0% (CI: 11.4–16.9%)
Among LVO	86/328 (26.2%)	N/A	26.2% (CI: 21.5–31.3%)
Among non-LVO	4/315 (1.3%)	N/A	1.3% (CI: 0.3–3.2%)
OR, LVO vs. non-LVO	4.09 (2.5–6.68)	N/A	4.09 (2.5–6.68)
Fraction due to LVO	86/90 (95.6%)	N/A	95.6% (CI: 89.0–98.8%)

*N/A: not available*.

Data on mortality within 3–6 months was only available from one study. Mortality at 6 months was higher after LVO than non-LVO cerebral ischemia, 26.2% vs. 1.3%, OR 4.1 (CI 2.5–6.6), *p* < 0.0001. Consequently, while LVO ischemic events accounted for 51.0% of all acutely presenting cerebral ischemia, they accounted for 95.6% (89.0–98.8%) of all poststroke mortality.

The literature search yielded the following values for inputs into the equation determining the proportion of acutely presenting cerebral ischemia patients treatable under current guidelines with ET. The proportion of AIS with ED arrival within 6 h was projected at 74.6%, based on national US registry data (15). Patients within 60 min ambulance travel time to an endovascular center were projected at 86%, based on a US population geospatial system optimization model (16). The rate of freedom from contraindications to ET among patients arriving within 7 h was projected at 86%, based on observed ineligibility rates in EXTEND-IA (17) including patients with poor pre-morbid function, large ischemic core, and poor venous access. From these inputs, it is projected that among all acute LVO-AIS patients, 55.2% are treatable within 8 h. In addition, these treatable LVO-AIS patients represented 21.4% of acutely presenting patients, but accounted for 34% of all poststroke combined dependence and death and 52.8% of all poststroke mortality (Table S2 in Supplementary Material).

## Discussion

This study confirms that LVOs contribute disproportionately to severe functional outcomes after AIS. LVOs cause a little more than one-third of acutely presenting acute cerebral ischemia events, but three-fifths of poststroke dependence and death and more than nine-tenths of poststroke mortality. This heightened impact amplifies the potential of ET to lessen the population-level burden of adverse stroke outcomes. In an optimized regional stroke-care system, one-fifth of all acutely presenting ischemic stroke patients are treatable with ET up to 8 h after onset, and, in the absence of treatment, these patients account for one-third of all combined dependence and death and one-half of all 3-month mortality after acutely presenting AIS.

These findings have implications for design of regional stroke-care system, indicating that optimizing access to ET will exert benefits at the population level in reducing poststroke dependency and death that substantially exceed simple treatment rates. We confined our review to studies performed entirely or largely in the era preceding modern thrombectomy devices, permitting assessment of contributions of LVO-AIS to functional outcomes not confounded by highly effective reperfusion interventions. We are not aware of prior reports systematically analyzing the contribution of LVO-AIS to severe poststroke outcomes after AIS. Our findings are consistent with prior estimates of the proportion of all ischemic strokes treatable by ET (4, 5, 18, 19). Our estimates focused on early presenting patients (within 24 h of onset), while prior estimates additionally included patients presenting subacutely to hospitals beyond 24 h or not presenting to hospitals at all with entirely outpatient work-ups. Such late-presenting patients are more likely to have minor, non-LVO ischemic strokes and relatively low rates of poststroke dependence and death than early arriving patients.

While we projected an optimized regional stroke-care system, key elements of current study findings apply broadly regardless of how care is geographically organized. The proportion of patients who are actually treated with ET within 8 h may vary from region to region, but the resulting impact of ET on poststroke combined dependence and death and poststroke mortality always will be disproportionately higher than the raw treatment rate.

This study has limitations. First, the two investigations meeting study criteria differed in some respects, including recruitment of only ischemic stroke (13) or both ischemic stroke and transient ischemic attack (11); recruiting patients presenting within 9 h vs. within 24 h of onset; and performance in Europe vs. North America. Second, the studies showed moderate heterogeneity, likely in part due to these different aspects; accordingly, we used a random-effect model in deriving pooled estimates. Third, the studies were from select cities in North America and Europe—studies of broader geographic and race–ethnic populations are desirable. Fourth, the rates of vessel patency to assess the mortality outcomes were available from a single study. Finally, it should be acknowledged that our analysis was derived from pooled model-based estimates from two studies, and further studies are needed to corroborate our results.

## Conclusion

As LVOs cause not only more than one-third of acutely presenting ischemic strokes but also three-fifths of poststroke dependence and death and more than nine-tenths of poststroke mortality, further dissemination of ET may have substantial benefit on population health.

## Author Contributions

KM: study concept and design, acquisition of data, analysis and interpretation, and critical revision of the manuscript for important intellectual content. JG: analysis and interpretation and critical revision of the manuscript for important intellectual content. JS: study concept and design, study supervision, and critical revision of the manuscript for important intellectual content.

## Conflict of Interest Statement

The authors declare that the research was conducted in the absence of any commercial or financial relationships that could be construed as a potential conflict of interest.
