# Supplementary material for: Ischemic Strokes Due to Large-Vessel Occlusions Contribute Disproportionately to Stroke-Related Dependence and Death: A Review
Source: Front Neurol. 2017 Nov 30;8:651. doi: 10.3389/fneur.2017.00651 (PMC5715197; doi:10.3389/fneur.2017.00651)
Supplement: Supplementary file 1 [file Data_Sheet_1.DOC]

Supplementary Material

**Ischemic Strokes Due to Large Vessel Occlusions Contribute Disproportionately to Stroke-Related Dependence and Death**

Konark Malhotra, MD ^1^; Jeffrey Gornbein, DrPH^2^; Jeffrey L. Saver, MD, FAHA, FAAN, FANA^3^

**Address for correspondence:**

Konark Malhotra, MD

West Virginia University Charleston Division

415 Morris Street, Suite 300

Charleston, WV 25301

Tel: 412-758-0737

Fax: 304-388-6445

Email: [Konark.malhotra@yahoo.com](mailto:Konark.malhotra@yahoo.com)

**Supplementary Data**

# Supplementary Figures and Tables

## Supplementary Figures

**Figure S1.** Study Search and Selection

Identification

Eligibility

Included

181 studies identified on MEDLINE, PubMed, Scopus databases

2 studies selected for final review

Total 8 studies were excluded on the basis of full text:

4; not consecutive AIS patients

1; lack of deficit severity criteria

2; based on clinical outcome association with severity and vessel occlusion

1; cohort <50 patients

Smith et al

187 studies excluded on the basis of the title and the abstract

10 studies selected

16 studies selected from the references of the pertinent articles

Total 197 studies reviewed

Van Seeters et al

**B) Supplementary Tables**

**Table S1.** Quality assessment of the included studies

|  | **Smith et al ^1^** | **Van Seeters et al ^2^** |
| --- | --- | --- |
| Exposed cohort representativeness | 1b | 1a |
| Non exposed cohort selection | 1 | 1 |
| Exposure Ascertainment | 1 | 1 |
| Absence of outcome at baseline | 1 | 1 |
| Comparability of cohorts | 2 | 2 |
| Outcome Ascertainment | 1 (a minus) | 1a |
| Long enough follow up | 1 | 1 |
| Follow up adequacy | 1 (b minus) | 1 |
| **Total score** | **9** | **9** |

**Table S2.** Fraction of AIS Outcomes occurring from Thrombectomy-Treatable Large Vessel Occlusions

|  | **Fraction Due to LVO** | **Fraction Due to LVO Treatable by Thrombectomy*** |
| --- | --- | --- |
| All Acute Cerebral Ischemia | 38.7% (CI: 21.8-55.7%) | 21.4% |
| Disability and Death (mRS 3-6) due to Acute Cerebral Ischemia | 61.6% (CI: 41.8-81.3%) | 34% |
| Death due to Acute Cerebral Ischemia | 95.6% (CI: 89.0-98.8%) | 52.8% |

*Values in this column obtained by multiplying values in second column by the proportion of all LVO AIS estimated to be treatable within 8h of onset = 55.2% (see text)

**Supplemental Citations**

1. Smith WS, Lev MH, English JD, Camargo EC, Chou M, Johnston SC, et al. Significance of large vessel intracranial occlusion causing acute ischemic stroke and tia. *Stroke; a journal of cerebral circulation*. 2009;40:3834-3840

2. van Seeters T, Biessels GJ, Kappelle LJ, van der Schaaf IC, Dankbaar JW, Horsch AD, et al. The prognostic value of ct angiography and ct perfusion in acute ischemic stroke. *Cerebrovascular diseases (Basel, Switzerland)*. 2015;40:258-269
